# A unique mechanism of successful fertilization in a domestic bird

**DOI:** 10.1038/srep07700

**Published:** 2015-01-09

**Authors:** Tomohiro Sasanami, Shunsuke Izumi, Naoki Sakurai, Toshifumi Hirata, Shusei Mizushima, Mei Matsuzaki, Gen Hiyama, Eriko Yorinaga, Takashi Yoshimura, Kazuyoshi Ukena, Kazuyoshi Tsutsui

**Affiliations:** 1 Department of Applied Biological Chemistry, Faculty of Agriculture, Shizuoka University, 836 Ohya, Shizuoka 422-8529, Japan; 2 Department of Mathematical and Life Sciences, Graduate School of Science, Hiroshima University, Higashi-Hiroshima 739-8526, Japan; 3 Department of Environmental Dynamics and Management, Graduate School of Biosphere Science, Hiroshima University, Higashi-Hiroshima 739-8528, Japan; 4 Graduate School of Bioagricultural Sciences, Nagoya University, Furo-cho, Chikusa-ku, Nagoya 464-8601, Japan; 5 Section of Behavioral Sciences, Graduate School of Integrated Arts and Sciences, Hiroshima University, Higashi-Hiroshima 739-8521, Japan; 6 Department of Biology and Center for Medical Life Science, Waseda University, 2-2 Wakamatsu-cho, Shinjuku, Tokyo 162-8480, Japan

## Abstract

Fertilization is an indispensable step for formation of a zygote in sexual reproduction, leading to species survival. When mating occurs, sperm is transported to the female reproductive tracts via the seminal plasma (SP). SP is derived from male accessory sex glands and it plays pivotal roles for fertilization in animals. However, molecular mechanisms of SP or a fluid derived from male accessory sex glands for successful fertilization remain unclear. Here, we report that in male quail the cloacal gland (CG) produces prostaglandin F_2α_ (PGF_2α_) that contributes to successful fertilization. PGF_2α_, as well as the secretion of CG (CGS), induced vaginal contractions and caused the opening of the entrance of the sperm storage tubules, the structures responsible for the long-term sperm storage and fertilization. The removal of CGS from the male before mating reduced the fertility, but the supplementation of CGS or PGF_2α_ rescued the subfertility. We further showed that male CG contains glucose that is utilized as energy source for the intrinsic sperm mobility after transportation to female vagina. This mechanism, in concert with the excitatory effects of PGF_2α_ enables successful fertilization in the domestic bird.

Fertilization is of paramount importance to species survival and its success depends on ejaculated sperm traversing the female reproductive tract to reach the oocytes where fertilization occurs. During mating, sperm are transported to the female reproductive tract within a fluid medium, generally referred to seminal plasma (SP) secreted from rete testis, epididymis and accessory sex glands of male genital tract^1^. The studies on the SP in many species highlight pivotal roles of SP in successful fertilization, including augmentation of sperm motility, modification of female receptivity and behavior, decreasing immune responses against allogenic spermatozoa and enhancing sperm transport within the oviduct^2^,^3^,^4^. Most of the semen of murine rodent coagulates to form a copulatory plug, which reduces the fertilization success of rival males and prevents sperm loss by a backward flow^1^. SP contains signaling substances such as sex steroids, prostaglandins and glycoproteins including cytokines and growth factors^2^,^4^. These molecules bind to their respective receptors on the target cells of the female reproductive tract to modulate functions related to fertilization. For example, immune-regulatory molecules such as transforming growth factor β (TGFβ) and prostaglandin E, found in the SP, had been suggested to modulate female immune response for male antigen-specific tolerance^2^. Indeed, an earlier study demonstrated that mated female mice fail to reject skin grafts of paternal origin^5^. The direct effects of SP components on the sperm had also been suggested. A family of heparin-binding proteins in bovine SP, that are referred to as bovine seminal plasma proteins (BSPs), coat the sperm surface and enable sperm to bind to the oviductal epithelium to prolong sperm motile life span in the oviduct^6^. Prostaglandin F_2α_ (PGF_2α_), the acidic soluble substance, which is produced in the seminal vesicle of male mammals is detected in the SP of various animals, although its concentration varies among species^7^. Studies in boar suggest that this hormone is important for sperm transport to the site of fertilization in the oviduct probably due to the stimulation of uterine contractility^8^,^9^. Indeed, it is reported that exogenous PGF_2α_ added to boar semen improved conception and farrowing rates after artificial insemination^10^. In addition to the effects of PGF_2α_, it is reported in pigs that SP estrogens can trigger local uterine endometrium PGF_2α_ release and thus increase uterine activity^11^. Moreover, PGF_2α_ also thought to be involved in excessive sperm elimination from the uterus since polymorphonuclear neutrophils are activated by sperm to release PGF_2α_ via the cyclooxygenase pathway to cause contraction of smooth muscle to remove accumulated fluid including sperm in the lumen^12^. It is considered that PGF_2α_ might improve reproductive efficiency by enhancing sperm transport within female reproductive tract to the fertilization site in mammals as PGF_2α_ dose not increase any sperm motility parameter^13^. Although PGF_2α_ in the SP is generally recognized as an agent that contracts smooth muscles of female reproductive tract, molecular mechanisms of how the myometrial contraction by the action of PGF_2α_ improves sperm transport remain unclear.

In avian species, unique reproductive strategies, such as polyspermic fertilization and sperm storage in the oviduct, are employed for successful fertilization. Thus, the characterization of avian reproductive strategies will provide deeper understanding of the reproductive system of vertebrates. The male reproductive system in birds is quite different in morphology from that in mammals, as it lacks the prostate and seminal vesicles^14^. Mature spermatozoa travel down the vas deferens, which is expanded into a sac-like ending near the cloaca (Fig. 1a). At copulation, male cloaca is everted and applied to the opening of the oviduct, *i.e.* vagina (Fig. 1a, b). Male cloacal gland (CG), a unique structure present in the dorsal wall of the cloaca, is noticeable as the exocrine gland in galliform species^15^. Mature male quail produce foam as an accessory sexual fluid in the CG (Supplementary Movie 1). This foam, which is obviously different from a lymph-like fluid produced by roosters, drakes and male turkeys, but rather close to the secretion by mammalian accessory sex glands^16^, is ejected with sperm in the opening of the oviduct, *i.e.* vagina, during copulatory behavior^17^. Thus, the CG of mature birds has been thought to produce essential substance(s) implicated in fertilization. However, the chemical features of CG products that play a role for successful fertilization have yet to be characterized.

In this study, we first identified PGF_2α_ in the CG of male quail and subsequently demonstrated unique mechanisms of PGF_2α_ for successful fertilization after transportation with sperm to female vagina by copulation in quail. We further found that the CG contains glucose that acts as energy source for the intrinsic mobility of sperm after transportation to female vagina in this domestic bird.

## Results and Discussion

**Figure 1:**
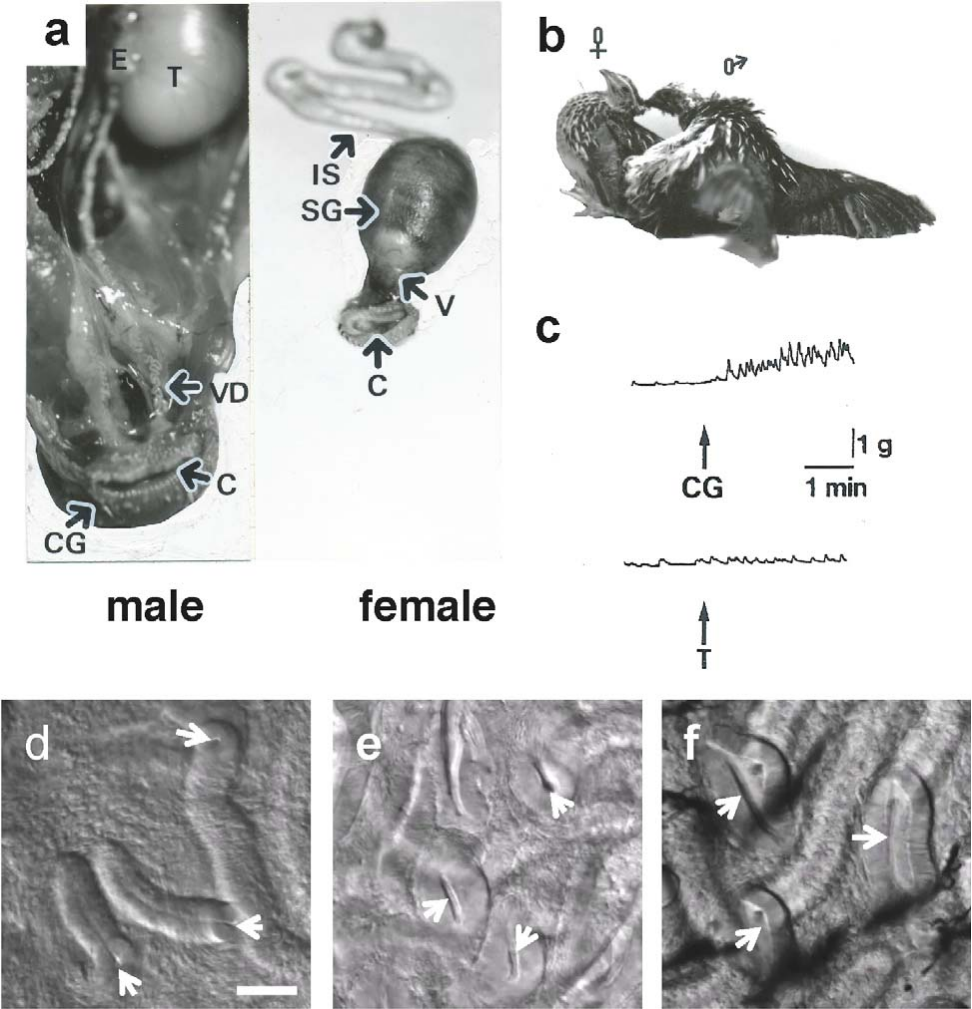
The reproductive system in the male and female Japanese quail. (a), Male, T. testis; E. epididymis; VD. vas deferens; C. cloaca; CG. cloacal gland. Female, IS. isthmus; SG. shell gland; V. vagina; C. cloaca. (b), The copulatory behavior of Japanese quail showing the cloacal contact movement. (c), Bioactivity of the crude extract derived from the cloacal gland (CG) or testis (T) on spontaneous contractions of the isolated female vagina (V). The upward arrow indicates application of the extract to the tissue. (d–f), Microscopic observation of the entrance of sperm storage tubules (SST). Mature females were intra-vaginally injected with (d), 30 μl phosphate-buffered saline (PBS), (e), cloacal gland secretion (CGS) or (f), 2 × 10^−9^ M prostaglandin F_2α_ (PGF_2α_). After 10 min injection, the mucosa of utero-vaginal junction was isolated and observed under light microscope. Arrows indicate the entrance of SST. Bar = 50 μm.

**Figure 2:**
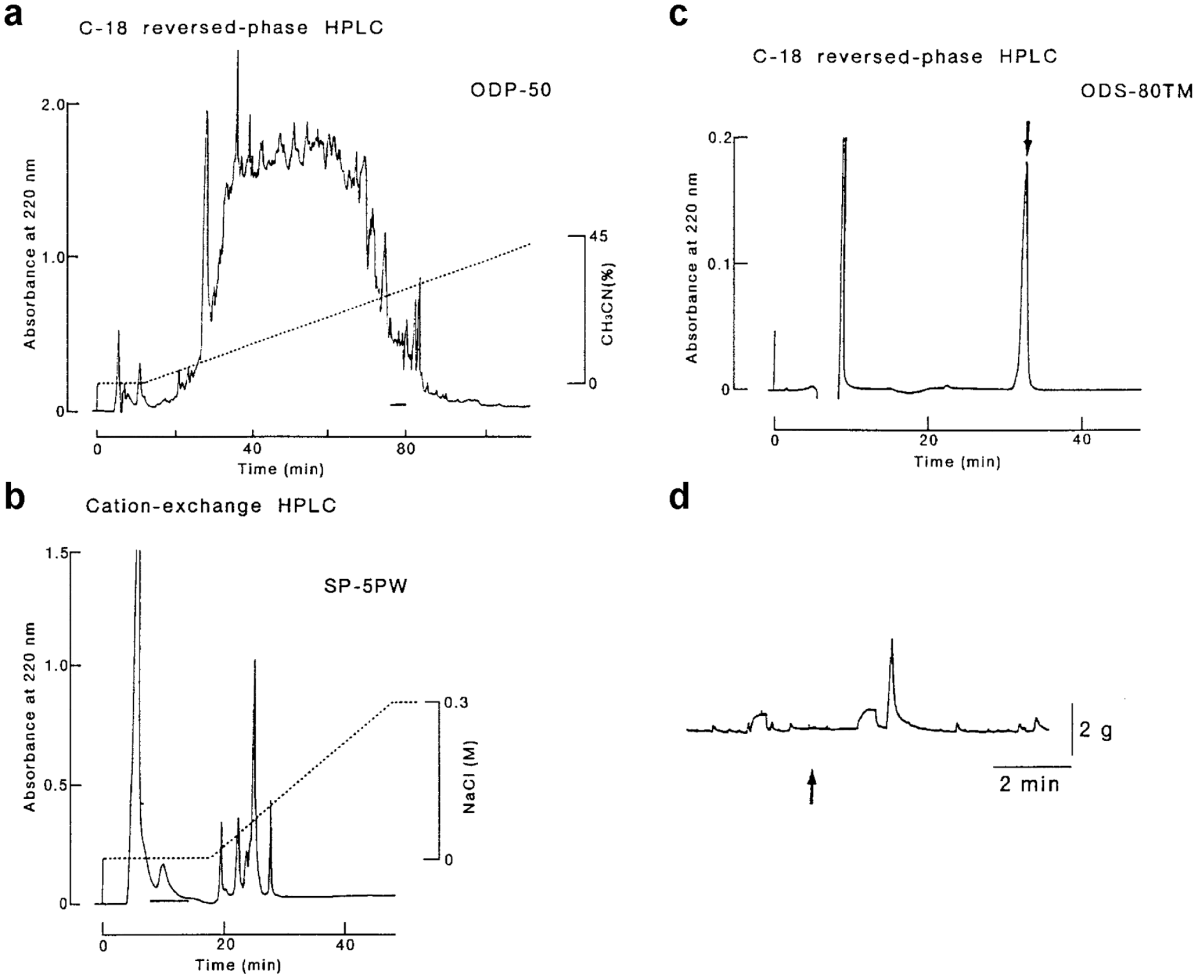
HPLC profile of the retained material (RM) of disposable C-18 reversed-phase cartridges on a reversed-phase column. (a), The RM loaded onto the column was eluted with a linear gradient of acetonitrile (ACN) and collected in 50 fractions of 2 ml each. Aliquots (20 μl) of each fraction were evaporated to dryness, dissolved in the physiological saline (pH 7.3) and applied to the bioassay. The bioactive fractions were indicated by the horizontal bar. (b), HPLC profile of the bioactive fractions (in panel a) on a cation-exchange column. Elution was performed in a linear gradient of NaCl in PB (pH 6.8) and eluents were collected in 1-ml fractions. The bioactivity was detected in the flow through fractions (horizontal bar). (c), Final purification of the bioactive substance (0.21 μmoles) by HPLC using a reversed-phase column. Isocratic elution with 36% ACN at a flow rate of 0.3 ml/min. (d), Bioactivity of the isolate on spontaneous contractions of the isolated female vagina. The upward arrow indicates application of an aliquot (2.5 × 10^−11^ M) of the isolate.

In the first experiment, we found that a crude extract derived from the CG of two males potentiates spontaneous contractions of the female vagina (Fig. 1c). In contrast, the testicular extract did not induce such a significant change (Fig. 1c). Likewise, injections of cloacal gland secretion (CGS) into the vagina caused the opening of the entrance of sperm storage tubules (SST) (compare Fig. 1, d and e), the specialized simple tubular invaginations responsible for the long-term sperm storage and fertilization in birds^18^. These results prompted us to identify the active components responsible for the stimulation of vaginal contractions. By using a series of C-18 reversed-phase columns (Fig. 2a) and a cation-exchange column (Fig. 2b) of the high-performance liquid chromatography (HPLC) system (Supplementary Fig. S3), we purified a bioactive substance from the extract of the CG of 100 males showing a single peak on the final reversed-phase column under an isocratic condition (Fig. 2c). The spectroscopic data of the isolated bioactive substance (Supplementary information) identified prostaglandin F_2α_ (PGF_2α_) as compared with the spectra of authentic specimens, such as PGF_2α_, (15*R*)-PGF_2α_ and (13*Z*, 15*R*)-PGF_2α_^19^. The calculated amount of the isolated substance (PGF_2α_) from the tissues of 100 males was 0.21 μmols. An excitatory effect of this substance (2.5 × 10^−11^ M) was detected in the vaginal bioassay (Fig. 2d). In addition, intra-vaginal injection of 2 × 10^−9^ M PGF_2α_, which is equivalent to the PGF_2α_ concentration in the CGS (2.1 ± 0.25 × 10^−9^ M, mean ± SEM, n = 3), successfully opened the entrance of SST (Fig. 1f). Because the effective dose of PGF_2α_ coincided with that in the CGS, the observed effects of PGF_2α_ are considered as physiological actions. Effects of PGF_2α_ on the spontaneous contractions of the female vagina was inhibited by pretreatment of female vagina with AL 8810, a selective antagonist for the receptor of PGF_2α _(PGF_2α_-R)^20^ (Supplementary Fig. S1).

**Figure 3:**
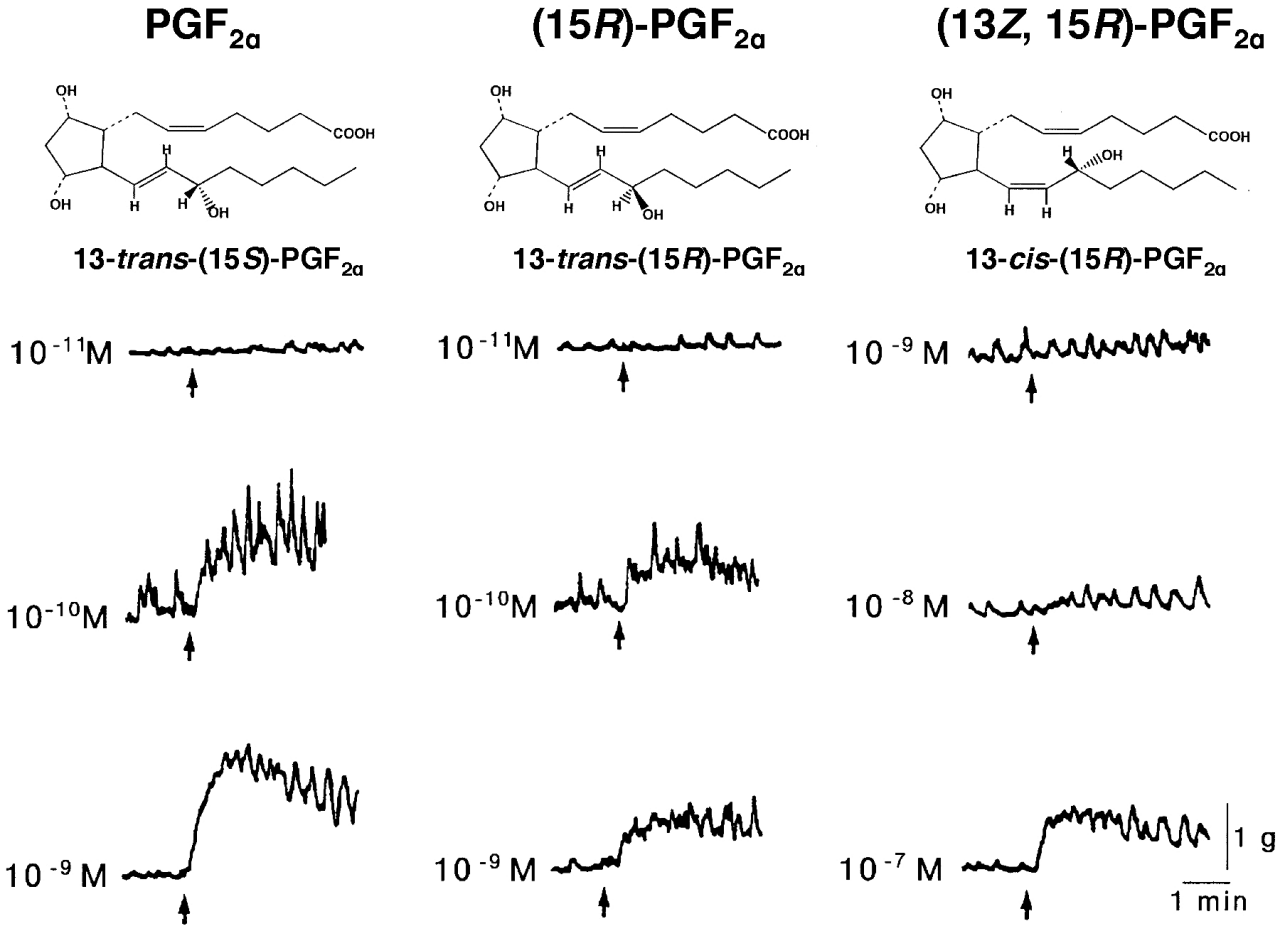
Bioactivities of PGF_2α_, (15*R*)-PGF_2α_ and (13*Z*, 15*R*)-PGF_2α_ on spontaneous contractions of the isolated female vagina. The upward arrow indicates application of each stereoisomer.

**Figure 4:**
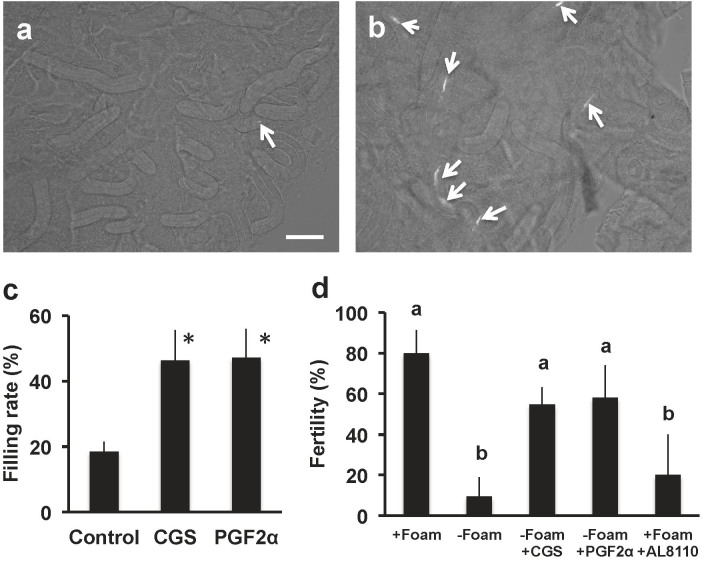
Effects of cloacal gland secretion (CGS) and PGF_2α_ on the fertilization of Japanese quail. Female quail were treated with (a), vehicle alone, or (b), CGS. After 10 min, the Hoechst 33342-stained sperm were artificially inseminated. The utero-vaginal junction mucosa was isolated from the bird 1 h after the artificial insemination (AI), and SST was observed under fluorescence microscope. Arrows indicate the resident sperm in the SST. Bar = 100 μm. (c), The percentage of the SST containing sperm was calculated. The data are expressed as the mean ± SEM of 3 replicates. An asterisk indicates a significant difference, *P* < 0.05. (d), The female was mated with the male without treatment (+Foam), or the male in which foam was manually removed (−Foam). The female was intra-vaginally injected with CGS (−Foam +CGS) or 2 × 10^−9^ M PGF_2α_ (−Foam +PGF_2α_) and were allowed to mate with the male, which had his foam manually removed. The female of which was intra-vaginally injected 1 μM AL 8810 was mated with intact male (+Foam +AL8810). Eggs were collected daily for 8 days and examined for embryo development in order to confirm fertilization. Fertility was calculated and expressed as the mean ± SEM. 3–5 birds were used for each treatment. Values with different letters are significantly different, *P* < 0.05.

The bioactivity of an isolated substance that results in vaginal contractions was compared with those of PGF_2α_ analogues. Although all of the stereoisomers resulted in vaginal contractions, PGF_2α_ caused the greatest effect (Fig. 3). From these results, we hypothesized that PGF_2α_ in the CGS supports sperm uptake into the SST by opening the SST entrance and vaginal contractions. To test this hypothesis, we measured sperm filling rate in the SST of quail, which were received intra-vaginal injection of CGS or PGF_2α_ before artificial insemination (AI) of the washed sperm. As the results, both CGS and PGF_2α_ treatment increased SST sperm filling (Fig. 4, a, b, c). These results indicated that the male bird transports PGF_2α_ into the vagina at the time of mating, and this substance supports the sperm uptake into the SST in quail. Presence of PGF_2α_-R in the utero-vaginal junction (UVJ) where SST exists was also confirmed by gene-specific *in situ* hybridization (Supplementary Fig. S2a). Our semi-quantitative RT-PCR analysis indicated that PGF_2α_-R mRNA was expressed not only in the vagina and UVJ, but was also present in the uterus, isthmus, magnum and infundibulum of the oviduct (Supplementary Fig. S2b). This observation coincides with previous findings that PGF_2α_ derived from preovulatory follicles induced contractions of the oviduct and might also be involved in the process of egg transport and oviposition^21^. We predicted that paternal PGF_2α_, transported into the female vagina at the time of copulation does not reach to the uterus beyond the UVJ. This is because our previous findings in which Hoechst 33342 DNA dye injected into the vagina was able to stain the nucleus of the surface epithelium of the vagina and UVJ, but not those of the uterus^22^. From these findings, we predicted that PGF_2α_-R expressed in the vagina and UVJ would only be involved in sperm transport and uptake into the SST. The elucidation of the roles of PGF_2α_-R expressed in the uterus or other parts of the oviduct for sperm transport should be examined in the future studies.

Constitutive expression of PGF_2α_-R in the UVJ throughout the ovulatory cycle is advantageous because females can copulate with a male at any time (Supplementary Fig. S2c). In agreement with this assumption, sperm filling rate of the SST was constant irrespective of the timing of mating during the ovulatory cycle (Supplementary Fig. S2d). Our results demonstrated the fertility of the male whose foam was manually removed before mating was significantly decreased when compared to that of the intact male (Fig. 4d). Notably, the intra-vaginal injection of CGS or PGF_2α_ before mating recovered fertility of the subfertile male (Fig. 4d). The hatchability of the fertilized eggs obtained from the CGS- or PGF_2α_-treated animals was 100% (4 or 3 birds were used for CGS or PGF2α treatment, respectively). These results indicated that this treatment had no adverse effects on embryo development. In addition, intact male fertility was decreased when AL 8110 was intra-vaginally injected prior to copulation (Fig. 4d). These results indicate the physiological importance of PGF_2α_ in the CGS for successful fertilization. The inability of the male to fertilize without foam appears to be related to the absence of PGF_2α_ as the biological basis of sustained fertility in domestic birds is in their capacity for the sperm to reside in the SST^23^. The concentrations of PGF_2α_ found in the SP of turkey and rooster are reported to be 100–140 pg/ml and less than 100 pg/ml, respectively^24^,^25^. Those values are approximately 7 times lower than that of quail CG. Although we did not perform vaginal bioassay in turkeys or chickens, their SP PGF_2α_ levels were sufficient to induce the vaginal contraction of quail. The question of whether this unique mechanism is also functional in other birds remains to be solved.

After natural mating, the ejaculated sperm are deposited into the vagina; however, it is reported that more than 80% of the sperm are ejected from the vagina following mating^18^. This suggests that the vagina is the primary sperm selection site in avian species. It is also reported that less than 1% of the sperm that are inseminated into the vagina are able to enter the SST^26^, and the resident sperm in the SST are thought to participate in the subsequent process of fertilization. In addition to the vaginal rejection of the sperm, the oviductal immune system may also affect the survivability of the sperm since the sperm are recognized as foreign bodies in the oviduct. Immunocompetent cells for acquired immunity (*i.e.* macrophages, antigen-presenting cells expressing MHC class II, CD4^+^, CD8^+^ T cells and premature B and plasma cells) are reported to localize in the oviduct mucosal tissue in sexually matured chickens to protect the tissues from infection^27^,^28^. A significant increase in the number of lymphocytes as well as antigen-presenting cells expressing MHC class II in the stroma of UVJ in low fertility hens were observed^29^. Das *et al.* demonstrated that the resident sperm in the SST of a fertile bird are protected from immune responses by TGFβ-mediated suppression of anti-sperm immunoreactions presumably by suppressing the proliferation of T- and B-lymphocytes^30^. Thus, the elimination of anti-sperm immune responses is one of the factors responsible for sperm maintenance in the SST. Although prostaglandin E was reported to induce oviposition in quail^25^, the immune-tolerance systems against male antigens that have been reported in the mammalian SP^2^ are not found in birds. Similarly, evidence for another sperm protection system in mice was recently published by Kawano *et al*. who observed that mice lacking seminal vesicle secretion 2 (SVS2) protein are infertile and their sperm are killed by uterus-derived cytotoxic factors^31^. They showed that SVS2 protein coats sperm surface and protects the sperm from the attack by the uterus. These views suggest that the SST provides a shelter for the sperm from the female attack. Importantly, the transport of PGF_2α_ at the time of mating is considered as a natural mechanism protecting the sperm from the rejection or killing by the female reproductive tract. Thus, the successful sperm uptake into the SST with the aid of PGF_2α_ action appears to be a key event for the achievement of fertilization in birds.

It is reported that CGS possesses the potency to augment sperm motility^32^,^33^, however, we failed to detect such stimulatory effects when the physiological concentration of PGF_2α_ was added to the sperm extender, indicating that PGF_2α_ does not participate in the enhancement of sperm motility in quail. To explore the machinery of how CGS contributes to the enhancement of sperm motility, free sugar analysis of a water-soluble phase of tissue homogenates of the CG was further carried out with the HPLC and gas chromatography (GLC) systems. The water-soluble extract of the CG contained free sugars, such as glucose, galactose and fructose (Supplementary Table S1). However unlike the CG, the testicular extract was completely devoid of glucose. Both the total amount and the concentration of fructose were lower than those of glucose and galactose in the CG (Supplementary Table S1). The present determination showing a significant level of glucose in the CG is in agreement with previous findings in which the production of the foamy material results from the production of carbon dioxide and hydrogen through bacterial action on glucose^34^. Accordingly, it is probable that glucose in the CGS is ejected as a foamy material accompanying with sperm, which may be utilized as energy source for sperm mobility. This mechanism, in concert with the excitatory effects of PGF_2α_, enables successful fertilization in quail. Our results provide a novel biological insight into the understanding of how the CG contributes to fertilization in a domestic bird.

## Methods

### Animals

Male and female Japanese quail, *Coturnix japonica*, 8–20 weeks of age (Motoki Corporation) maintained individually under a photoperiod of 14-h light (L): 10-h dark (D) (lights went on at 05:00) with *ad libitum* access to water and a commercial diet (Motoki Corporation) were used for experiments. All experimental procedures for the care and use of animals were carried out in accordance with approved guidelines of the Animal Care Committees of Shizuoka University and Waseda University.

### Extraction and purification of bioactive substance

Excised cloacal gland (CG) tissues (ca. 70 g wet weight) of 100 male quail were immediately frozen in liquid nitrogen. The frozen tissues were pulverized, boiled for 15 min in 4% acetic acid and homogenized by a Polytron. The homogenate was centrifuged at 10,000 × *g* for 40 min at 4°C and the supernatant fluid was evaporated to dryness. The dried extract was dissolved in 0.1% trifluoroacetic acid (TFA) and then forced through disposable C-18 cartridges (Mega Bond-Elut, Varian). The retained material (RM) eluted with 50% methanol was concentrated and filtered through a membrane (0.45 μm; Dismic-13cp, Advantec). The filtrate was subjected to the HPLC system (TRY ROTAR VI, Jasco) according to our previous methods^35^,^36^,^37^. In brief, the RM of disposable C-18 cartridges was loaded onto a C-18 reversed-phase column and eluted with a 120-min linear gradient of 0–60% acetonitrile (ACN) in 0.1% TFA (pH 2.2) at a flow rate of 1 ml/min. The bioactive fractions were then applied onto a cation-exchange column and eluted with a 70-min linear gradient of 0–0.7 M NaCl in 10 mM phosphate buffer (PB; pH 6.8) at a flow rate of 0.5 ml/min. The bioactive fractions were re-chromatographed by the other C-18 reversed-phase column with a linear gradient of 28–43% ACN in 0.1% TFA. Final purification was performed with the same column under an isocratic condition with 36% ACN at a flow rate of 0.3 ml/min. The purification scheme is presented in supplementary Fig. S3.

### Bioassay for vaginal contractions

The bioactivity of an extract and each HPLC fraction was examined by monitoring the effects on spontaneous contractions of the vaginal oviduct. For the bioassay, the vaginal oviduct of female quail was excised and cut transversely with 5–10 mm length. Both ends of the excised vagina were tied with two cotton threads, one being connected to the bottom of an experimental chamber and the other to a force-displacement transducer (NEC San-ei Instruments). The chamber was filled with 10 ml physiological saline (pH 7.3), which was bubbled with air and incubated at 37–39°C. The test substance dissolved in 0.1 ml saline was injected into the chamber.

### Structure determination of bioactive substance

Mass spectrometry (MS) analyses were performed on a JEOL JMS SX-102 equipped with a xenon beam generating system as a FAB-ion source at 10 kV of accele voltage by use of glycerol as a matrix. ^1^H NMR spectra were observed with a JEOL GSX-500 spectrometer at 500 MHz in CDCl_3_ using tetramethylsilane as an internal standard. The spectroscopic data of the isolated substance were compared with those of authentic specimens. Based on the results of structure analyses, the substance was identical with PGF_2α_. Therefore, the characterized native substance was further compared with PGF_2α_ with regard to behavior on HPLC.

### Semen collection and artificial insemination

Ejaculated semen was obtained from male quail during mating prior to ejaculation according to the procedure of Kuroki and Mori^38^. Semen obtained from two males was suspended in Hanks' balanced salt solution (HBSS) supplemented with 0.5 μM Hoechst 33342. The concentrations of sperm were measured with a hemocytometer. The ejaculates were washed two times with HBSS with repeated centrifugation at 800 × g for 3 min, and the final pellet was used for artificial insemination (AI).

### Fertility assay

The CGS was obtained by centrifugation of isolated foam at 20,000 × *g* for 10 min. PGF_2α_ level in the CGS was measured by ELISA kit (Cayman). The CGS or PGF_2α_ dissolved in PBS (1 ng/ml) was intra-vaginally injected (30 μl/bird). To observe the effects of AL8110 (Cayman), the chemical dissolved in dimethyl sulfoxide was diluted with PBS (1 μM), and intra-vaginally injected before copulation. The concentration of AL8810 was adopted from Griffen et al.^20^. After 10 min, the female was received AI or allowed to copulate with the male. Eggs were collected everyday and fertility was confirmed by the development of a blastoderm. In addition, some eggs were incubated at 37°C with rocking at a 30° angle until hatching to assess the hatchability.

### Observation of SST

The UVJ was isolated as described previously^39^. After washing with PBS, the specimens were fixed with 3.7% formaldehyde solution, and the fixed UVJ was cut into small pieces with scissors, mounted in glycerol and examined under a fluorescence microscope with a 20 x objective^39^ (BX 51, Olympus Optics). The total number of SST and the number of SST filled with sperm were counted, and the filling rate (%) of SST was calculated.

### *In situ* hybridization

The frozen sections of the isolated UVJ were prepared for *in situ* hybridization, as described elsewhere^40^. The antisense 45-mer oligonucleotide probes for the receptor for PGF_2α_ (5′- CATGGTTAGGAGACGAGAGGCAGCGCTCAACACATTTCAGCAGCC -3′, 5′- GCGATGGCTCCGTTGATGAGGTGGCCAAAGAGATCAGTGATGACC -3′, 5′- GGCTTGGTGACTCCAATGCAACGTTCAACAGCCATCACGCTGCCC -3′) were labeled with [33P] dATP (NEN Life Science Products) using terminal deoxyribonucleotidyl transferase (Gibco, Frederick). Hybridization was performed overnight at 42°C. Washing was performed twice at room temperature for 30 min and at 55°C for 40 min. After washing, the slides were exposed imaging plate (Fujifilm) and detected with BAS-2000 (Fujifilm).

### RT-PCR

Total RNA was extracted from the oviductal tissues with a commercial kit, RNAiso (Takara Biomedicals), according to the manufacturer's instructions. Messenger RNA was isolated using an oligotex-dT30 mRNA purification kit (Takara Biomedicals), according to the manufacturer's instructions, and was reverse transcribed using a ReverTra Ase kit (Toyobo). PCR amplification was performed using specific primer sets for the receptor for PGF_2α_ (sense: 5′- CCAACAGTCTCGCAATAGCA - 3′; antisense, 5′- AAAGTGGGCACAAACCAAAG- 3′). In parallel, primers (sense: 5′- GACGAAGACGGTGAAGAAGG-3′; antisense, 5′-CTTGGTGTCTGGGTCCACTT-3′) for quail S17 ribosomal protein^41^ were used as an internal control. For non-RT control, mRNA of UVJ was treated in a same way except to replace the reverse transcriptase with water. The products were analyzed on a 1.0% agarose gel stained with ethidium bromide and visualized under UV transillumination. The intensity of the bands was measured with NIH Image ver. 1.61.

### Free sugar analysis

For the measurement of free sugar contents, 1 ml homogenate of the CG was first boiled in deionized water for 10 min, and then 4 ml ethanol was added to the homogenate. Supernatant was obtained by centrifugation (1,000 × *g*, 10 min) and dried at 50°C with a stream of filtered air. Dried materials were dissolved in 1 ml distilled water, and then filtered through a membrane (0.45 μm; Gelman). Sample solution (0.5 ml) was introduced into the HPLC system (model 307, Gilson) equipped with a pulsed amperometric detector (Dionex). The column (CarboPac PA-1, Dionex) was eluted with 0.16 M NaOH at a flow rate of 1 ml/min. Fructose was eluted at 4.3 min. The other half sample solution was reduced for 1 h at room temperature by the addition of 0.5 ml NaBH_4_ (20 mg/ml) in 2 M NH_4_OH containing 300 μg inositol as an internal standard. Reduced monosaccharides were acetylated for 15 min at room temperature by the addition of 200 μl acetic anhydride and 50 μl 1-methylimidazole as a catalyst^42^. Acetylated monosaccharides were analyzed by gas chromatography (GLC; GC-7A, Shimadzu) with a capillary column (SP-2440, Supelco).

### Statistical analysis

Results were expressed as the mean ± SEM. The significance of differences between the groups was evaluated by one-way ANOVA followed by the Duncan's Multiple Range test or Student's *t* test (only Table S1). The differences were considered significant if *P* < 0.05.

## Author Contributions

T.S. and K.T. conceived and designed the study. T.S., S.I., N.S., T.H., S.M., M.M., G.H., E.Y., T.Y., K.U. and K.T. performed the experiments and wrote the paper. All authors approved the final manuscript.

## Supplementary Material

Supplementary Information Supplementary information

Supplementary Information Supplementary movie
