# Supplementary material for: A unique mechanism of successful fertilization in a domestic bird
Source: Sci Rep. 2015 Jan 9;5:7700. doi: 10.1038/srep07700 (PMC4287725; doi:10.1038/srep07700)
Supplement: Supplementary Information [file srep07700-s1.pdf]

A unique mechanism of successful fertilization in a domestic bird

Tomohiro Sasanami[1], Shunsuke Izumi[2], Naoki Sakurai[3], Toshifumi Hirata[2], Shusei Mizushima[1], Mei Matsuzaki[1], Gen Hiyama[1], Eriko Yorinaga[4], Takashi Yoshimura[4], Kazuyoshi Ukena[5], Kazuyoshi Tsutsui[6*]

[1]Department of Applied Biological Chemistry, Faculty of Agriculture, Shizuoka University, 836 Ohya, Shizuoka 422-8529, Japan.
[2]Department of Mathematical and Life Sciences, Graduate School of Science, Hiroshima University, Higashi-Hiroshima 739-8526, Japan.
[3]Department of Environmental Dynamics and Management, Graduate School of Biosphere Science, Hiroshima University, Higashi-Hiroshima 739-8526, Japan.
[4]Graduate School of Bioagricultural Sciences, Nagoya University, Furo-cho, Chikusa-ku, Nagoya 464-8601, Japan.
[5]Section of Behavioural Sciences, Division of Human Sciences, Graduate School of Integrated Arts and Sciences, Hiroshima University, Higashi-Hiroshima 739-8526, Japan.
[6]Department of Biology and Center for Medical Life Science, Waseda University, 2-2 Wakamatsu-cho, Shinjuku, Tokyo 162-8480, Japan.

*Corresponding to: Kazuyoshi Tsutsui, Department of Biology and Center for Medical Life Science, Waseda University, 2-2 Wakamatsu-cho, Shinjuku, Tokyo 162-8480, Japan. Email: k-tsutsui@waseda.jp

Supplementary Information

Table S1: Free monosaccharide composition from water soluble extracts of the cloacal gland and testis.
Figure S1: Effects of AL8110 on the $PGF_{2\alpha}$-induced spontaneous contractions of the isolated vagina.
Figure. S2: Expression of the receptor for $PGF_{2\alpha}$. in the utero-vaginal junction (UVJ).
Caption to Movie S1: Appearance of cloacal gland secretion (CGS).
Data of Structural analysis

Table S1. Free monosaccharide composition from water soluble extracts of the cloacal gland and testis.

Gland	Sugar content					
	Gal	Fru	Glc	Gal	Fru	Glc
	(μg/g)			(μg/gland)		
Cloacal gland	115	43	85	75	28	55
Testis	45	49	0*	136	148	0*

Free fructose (Fru) content in the extracts was determined by an HPLC system equipped with a pulsed amperometric detector, and free galactose (Gal) and glucose (Glc) were reduced and acetylated to alditol acetate form and were determined by GLC using inositol as an internal standard. *, Free glucose (Glc) in the testis was not detected by gas chromatography.

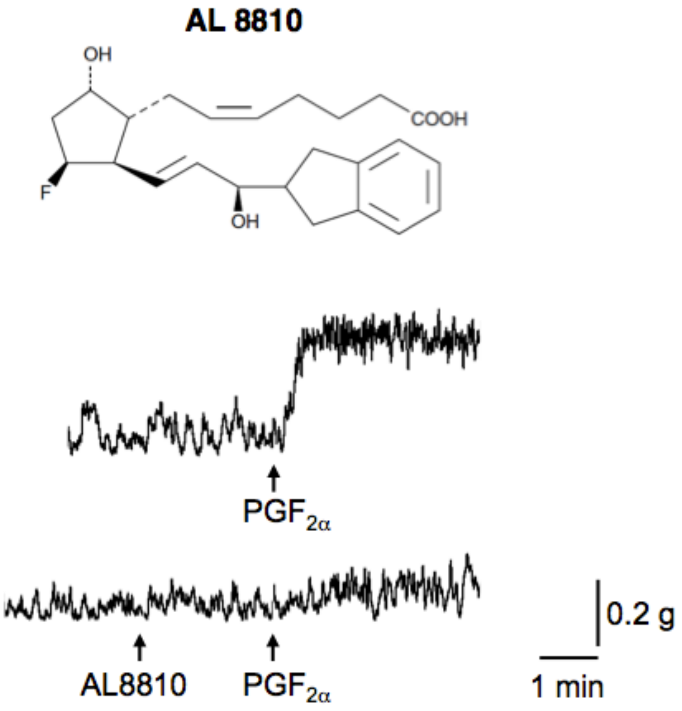

Figure S1 | Effects of AL 8110 on the PGF_2 –induced spontaneous contractions of the isolated vagina. The upward arrow indicated application of each chemical.

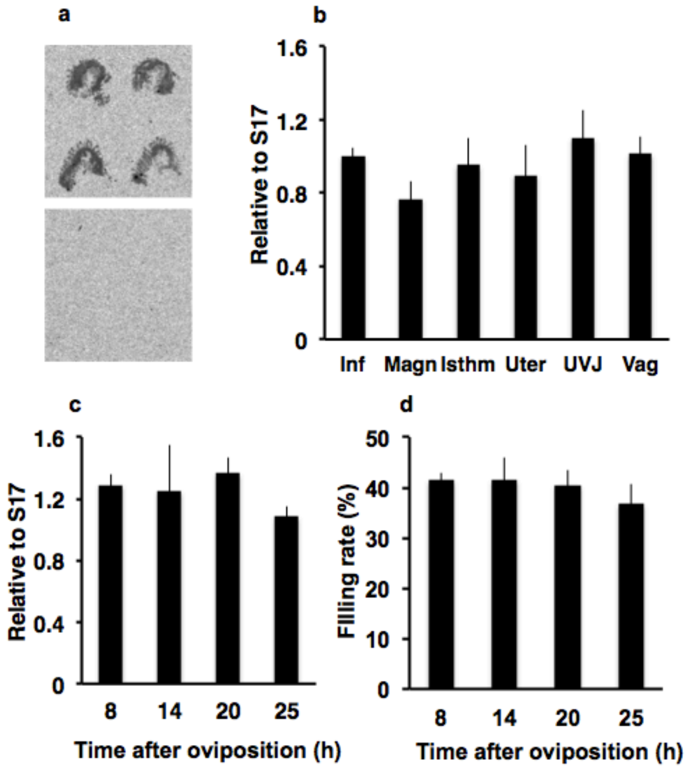

Figure S2 | Expression of the receptor for PGF$_{2\alpha}$. a, Autoradiograms of the UVJ sections after hybridization with ^{33}P-labeled antisense probe specific for the receptor for PGF$_{2\alpha}$ (upper panel) or sense probe (lower panel) are shown. Representative results of two experiments are shown (n = 2). **b,** RT-PCR analysis of the receptor for PGF$_{2\alpha}$. The infundibulum (Inf), magnum (Magn), isthmus (Isthm), uterus (Uter), UVJ or vagina (Vag) isolated from females at 14 h after egg-laying were processed for RT-PCR analysis using gene-specific primers. Data were normalized with S17 ribosomal protein and expressed as the mean ± SEM of three independent experiments. **c,** RT-PCR analysis of the receptor for PGF$_{2\alpha}$ during ovulatory cycle. The UVJ isolated from females at 8, 14, 20 or 25 h after egg-laying were processed for RT-PCR analysis using gene-specific primers. Data were normalized with S17 ribosomal protein and expressed as the mean ± SEM of three independent experiments. **d,** Comparison of sperm filling rate during ovulatory cycle. Females were mated at 8, 14, 20 or 25 h after egg-laying. The utero-vaginal junction mucosa was isolated from the bird, and SST was observed under fluorescence microscope. Sperm filling rate was calculated and expressed as the mean ± SEM. 3–5 birds were used within each treatment.

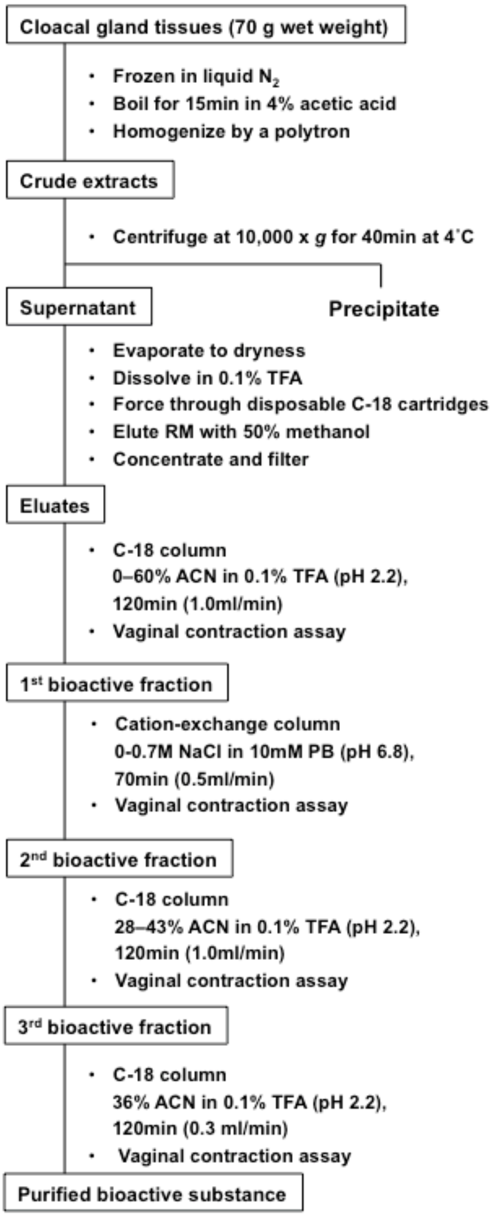

Figure S3 | Purification scheme of bioactive substance from cloacal gland extracts. TFA: trifluoroacetic acid , RM: retained materials, ACN: acetonitrile, PB: phosphate buffer.

Caption to Movie S1

Appearance of cloacal gland secretion (CGS).

The cloacal gland (CG) of male quail ejects CGS as a meringue-like foam by pushing lightly with the thumb and index finger.

Data of structural analysis

The MS, IR and 1H NMR spectra of the isolated bioactive substance were as follows: MS m/z 355 (M+1), 337 [(M-H2O)+1], 319 [(M-2H2O)+1] and 301 [(M-3H20)+1]; IR (Liq. film) υ 3400-3300 (OH) and 1708 cm-1 (COOH); 1H NMR δ 0.89 (3H, t, J=6.5 Hz, 20-H3), 4.02 (1H, m, 11-H), 4.22 (1H, q, J=6.2 Hz, 15-H), 4.25 (1H, dd, J=5.4 and 4.5 Hz, 9-H), 5.38 (1H, bq, J=7.5 Hz, 5-H), 5.53 (1H, bq, J=7.5 Hz, 6-H), 5.59 and 5.60 (2H, m, 13- and 14-H). The spectroscopic data of the isolated bioactive substance were found to be prostaglandin F2α (PGF2α) as compared with the spectra of authentic specimens, such as PGF2α, (15R)-PGF2α and (13Z, 15R)-PGF2α (Cayman Chemical).
